# Supplementary material for: Association between oral microbiome and seven types of cancers in East Asian population: a two-sample Mendelian randomization analysis
Source: Front Mol Biosci. 2023 Nov 21;10:1327893. doi: 10.3389/fmolb.2023.1327893 (PMC10702768; doi:10.3389/fmolb.2023.1327893)
Supplement: Supplementary file 1 [file DataSheet1.ZIP › Supplementary Materials/MR plots for tongue/tongue═╝/Pancreatic cancer/╥╚╧┘░⌐_╔╕╤í╜ß╣√/pheno.1387_to_pancreatic cancer_leave_one_out.pdf]

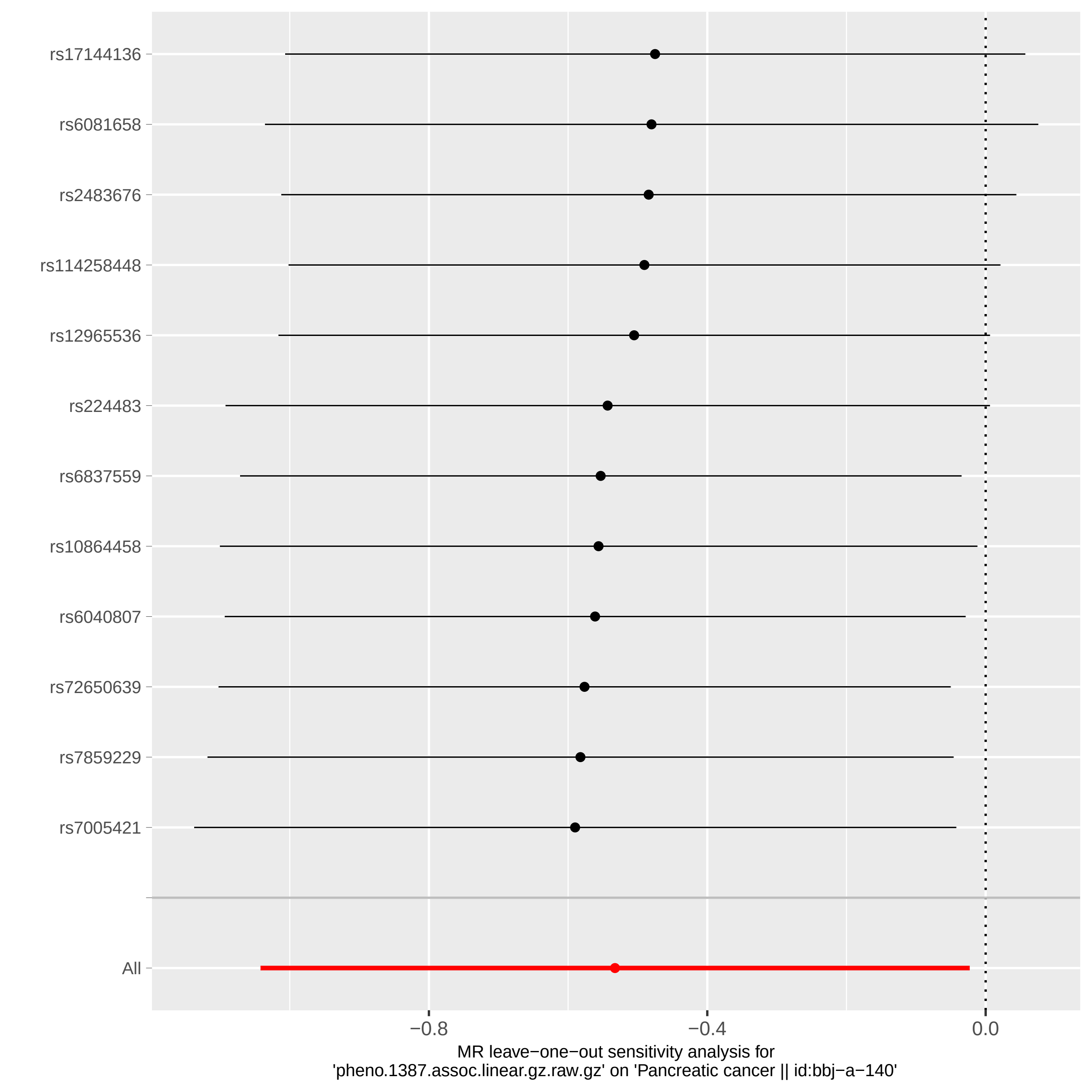
rs17144136
rs6081658
rs2483676
rs114258448
rs12965536
rs224483
rs6837559
rs10864458
rs6040807
rs72650639
rs7859229
rs7005421
All
−0.8
−0.4
0.0
MR leave−one−out sensitivity analysis for
'pheno.1387.assoc.linear.gz.raw.gz' on 'Pancreatic cancer || id:bbj−a−140'
